# Ultrasound Imaging versus Radiographs in Differentiating Periapical Lesions: A Systematic Review

**DOI:** 10.3390/diagnostics11071208

**Published:** 2021-07-03

**Authors:** Shankargouda Patil, Ahmed Alkahtani, Shilpa Bhandi, Mohammed Mashyakhy, Mario Alvarez, Riyadh Alroomy, Ali Hendi, Saranya Varadarajan, Rodolfo Reda, A. Thirumal Raj, Luca Testarelli

**Affiliations:** 1 Department of Maxillofacial Surgery and Diagnostic Sciences, Division of Oral Pathology, College of Dentistry, Jazan University, Jazan 45142, Saudi Arabia; dr.ravipatil@gmail.com; 2 Department of Restorative Dental Sciences, College of Dentistry, King Saud University, Riyadh 11451, Saudi Arabia; ahkahtani@ksu.edu.sa; 3 Department of Restorative Dental Sciences, College of Dentistry, Jazan University, Jazan 45142, Saudi Arabia; shilpa.bhandi@gmail.com (S.B.); dr.mashyakhy@gmail.com (M.M.); 4 Division of Endodontics and Orthodontics, University of Southern California, Los Angeles, CA 90007, USA; Marioa@usc.edu; 5 Department of Restorative Dental Sciences, College of Dentistry, Majmaah University, AlMajmaah 11952, Saudi Arabia; r.alroomy@mu.edu.sa; 6 Department of Radiology, Faculty of Medicine, Jazan University, Jazan 45142, Saudi Arabia; ahendi@jazanu.edu.sa; 7 Department of Oral Pathology and Microbiology, Sri Venkateswara Dental College and Hospital, Chennai 600130, India; vsaranya87@gmail.com (S.V.); thirumalraj666@gmail.com (A.T.R.); 8 Department of Oral and Maxillofacial Sciences, Sapienza University, University of Rome, 00161 Rome, Italy; luca.testarelli@uniroma1.it

**Keywords:** imaging, ultrasound, radiograph, periapical, lesions, diagnosis

## Abstract

Background: Ultrasonography is a non-invasive method of diagnosing periapical lesions while radiologic methods are more common. Periapical lesions due to endodontic infection are one of the most common causes of periapical radiolucency that need to be distinguished to help determine the course of treatment. This review aimed to examine the accuracy of ultrasound and compare it to radiographs in distinguishing these lesions in vivo. Methods: This review process followed the PRISMA guidelines. A literature search of databases (PubMed, Scopus, Embase, and Web of Science) was conducted without any restrictions on time. Articles available in English were included. The selection was done according to the inclusion and exclusion criteria. The QUADAS-2 tool was used to assess the quality of the studies. Results: The search provided a total of 87 articles, out of which, five were selected for the final review. In all the studies, ultrasound had higher accuracy in distinguishing periapical lesions. All the studies indicated a risk of bias, especially in patient selection. Conclusion: Within limitations, the study indicates that ultrasound is a better diagnostic tool to distinguish periapical lesions compared to radiographs but further studies with well-designed, rigorous protocols and low risk of bias are needed to provide stronger evidence.

## 1. Introduction

An area of bone resorption at the apex of a tooth is referred to as a periapical lesion and is most commonly seen as a periapical radiolucency [1]. In an infected root canal system, toxins from the canal reach periapical tissues and evoke an inflammatory response leading to resorption of hard tissues, periapical destruction, and the development of a periapical lesion [2,3]. Periapical lesions can aggravate systemic conditions, and conversely, individuals with systemic health problems may be prone to developing periapical lesions [4]. The presence of periapical lesions is significantly higher in diabetics [5]. Their prevalence has also been associated with coronary artery disease [6] and postmenopausal osteoarthritis [7]. 

Studies that examined the prevalence of periapical lesions, histologically, found that these are mainly periapical granulomas and radicular cysts that together make up nearly 90% of the periapical lesions [8,9,10,11]. The occurrence of periapical lesions is as high as 86% in some populations [4]. Recently, one study indicated that the prevalence of periapical lesions in different populations is not very different from that found earlier [4]. Another indicated that the occurrence of these lesions in the adult population, in both endodontically treated and untreated teeth, has increased over the past eight years [12]. 

It is important to distinguish periapical lesions as cysts or granulomas [13]. Granulomas are a mass of soft tissue with chronic inflammatory cells while cysts are epithelium-lined cavities with fluid, semi-solid, or cellular debris [14]. Cases that are refractory to non-surgical endodontic treatment are often associated with inflammatory cysts that need periapical surgery [15,16,17]. Both periapical granulomas and cysts are associated with infection of the root canal and a pre-operative knowledge of the lesion will help undertake necessary procedures, such as instrumentation beyond the apex, and advise the patient regarding the chances of resolution without surgical intervention. Only teeth with complete resolution of the periapical lesion can be used as abutments for restoration. However, a prolonged observation period to determine the outcome is not desirable because treated teeth must be restored in time to avoid failure of treatment [18].

Conventional radiographs, the most commonly available tool in the dental clinic, have been used to distinguish cysts and granulomas based on their appearance [19]. Recently, 3D radiographic imaging in the form of Cone Beam Computed Tomography (CBCT) has also been used to differentially diagnose periapical lesions from cysts [20]. CBCT was found to have better accuracy than radiographs in the diagnosis of periapical lesions but the studies do not justify its use regularly [21]. The differentiation of periapical lesions with radiologic modalities has shown results with varying accuracies [22,23,24]. These imaging modalities are invasive and not as accurate as is the gold standard—histopathological diagnosis [25,26]. The drawback of histopathology, however, lies in its invasiveness, time consumed, and the need for a specialized setup for diagnosis that is not available at every dental clinic. Other modalities such as ultrasonography (USG) and Magnetic Resonance Imaging (MRI) also permit differentiating cysts and granulomas [27,28,29]. They are non-invasive diagnostic methods that can be used in vivo. MRI, however, is associated with high costs, the need for expensive equipment, longer scan times for adequate resolution, and device-related artifacts [27,29,30,31,32].

Ultrasonography (USG) is a non-invasive, real-time imaging technique that uses ultrasound waves to differentiate and map an area where a loss or change in hard tissue architecture has occurred [32]. It has been successfully used in endodontics to image periapical lesions and also to distinguish cysts and granulomas [31,32]. In addition to the extent of the lesion, echography provides information regarding its contents and vascularity which can help distinguish endodontic and other maxillofacial bony lesions. It has, therefore, been suggested as a valuable diagnostic tool, one which can be used for routine imaging in the dental clinic [28]. However, certain drawbacks of this technique are mentioned in the literature: it can only be used when the cortical plate is eroded or the architecture of bone has changed and it does not distinguish between the types of periapical cysts [25,33].

This review, therefore, aimed to determine the diagnostic accuracy of imaging modalities of radiography, which is currently more common, in comparison to ultrasound imaging using histopathologic diagnosis as the gold standard. The focus question addressed by this review was: *Does ultrasound imaging provide better diagnostic results in differentiating endodontic lesions compared to conventionally used radiographic methods of imaging?*

## 2. Materials and Methods

This systematic review followed the guidelines provided in the Preferred Reporting Items for Systematic Reviews and Meta-Analysis (PRISMA) [34]. Literature searches of the relevant databases: PubMed, Scopus, Web of Science, and Embase, were conducted using the queries mentioned in Table 1, on 22 April 2021. There were no restrictions applied to the period of the search but only articles available in English as full texts were included.

The search results were exported to EndNote software online (Clarivate™, Sydney, Australia) from all databases. Duplicates were removed using the software. The study titles and their abstracts were then screened as per the criteria for inclusion and exclusion determined as follows:

Inclusion criteria: The PICOS criteria were used to determine inclusion for this review.

Population: Patients with symptoms of endodontic lesions or periapical radiolucencies.

Intervention: Ultrasound images for the diagnosis of periapical lesions and their differentiation.

Comparison: Radiographic images for the diagnosis of periapical lesions and their differentiation.

Outcomes: Correct identification of periapical lesion as cyst, granuloma, or a mixed lesion.

Studies: Comparative (prospective) diagnostic studies, randomized controlled trials, clinical trials.

Studies comparing the accuracy of diagnosis obtained with both techniques to a histopathological result, which is the gold standard, were included.

Exclusion Criteria: Narrative reviews, case reports, opinion pieces, conference abstracts, and letters to the editor, and articles that were not published in English were excluded.

The articles were screened by two reviewers separately. Any disagreements during the screening of articles were discussed with another reviewer for resolution. Studies identified during the screening were subject to a full-text examination separately by two reviewers. Disagreements were resolved, as before, by discussion. The studies found eligible for inclusion in the review were hand searched for references to additional studies. Prominent journals in related fields—Journal of Endodontics, International Endodontic Journal, Dentomaxillofacial radiology—were also searched for studies related to the topic. The studies finally selected for review were subject to the extraction of data relevant to the review question. Data was presented in a tabular form.

Risk of Bias Assessment: The Quality Assessment of Diagnostic Accuracy Studies (QUADAS-2) tool developed by both the University of York and the University of Amsterdam was used [35]. It has also been adopted and recommended for use for reviews on Diagnostic Test Accuracy (the Cochrane Collaboration) to assess the quality of included studies [36]. It is also recommended by the National Institute of Health Care and Excellence in the UK, and the Agency for Healthcare Research and Quality in the US. This tool has four domains that test the risk of bias and also the applicability of patient selection, the index test(s), the reference standard, and flow and timing. Each domain consists of signaling questions to help assist the judgment reached. In addition to the risk of bias, the tool also assesses the applicability concern of the studies to the review question pertaining to the first three criteria i.e., patient selection, index test, and the reference standard. This tool was used for the assessment of two index tests—Ultrasonography (Index test 1) and radiologic imaging (Index test 2). Histopathologic examination was considered the reference standard. The results were presented in the recommended tabular form.

## 3. Results

**Figure 1:**
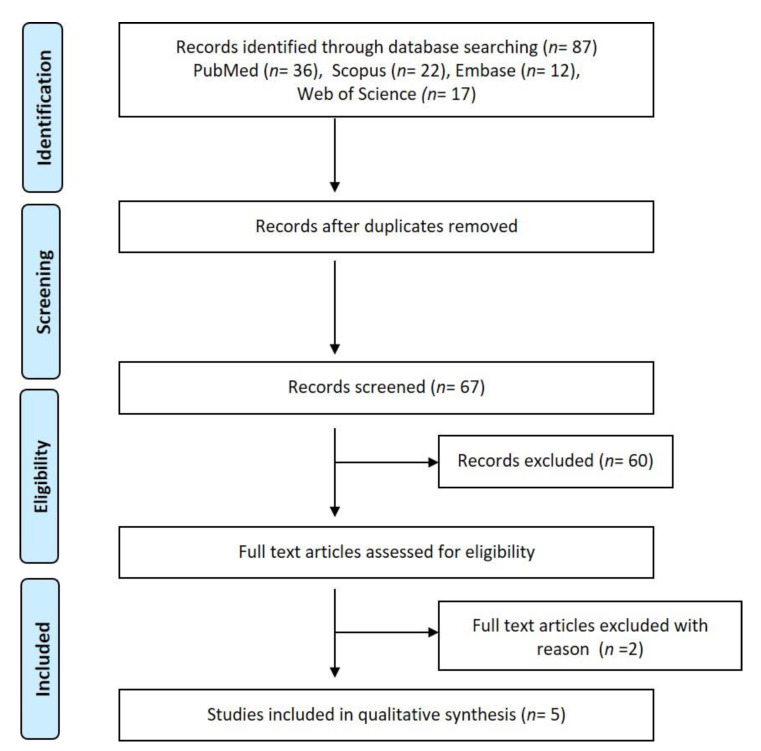
PRISMA flow diagram depicting the methodology of the review.

The initial literature search results provided 87 articles. From these, 20 were eliminated as they were duplicates and the remaining subject to screening. Both reviewers were in almost perfect agreement regarding the selection of articles (κ = 0.92). The discrepancy was resolved by discussion with a third reviewer and seven articles selected for reading the entire text. The flowchart for the selection process, according to PRISMA guidelines, is provided in Figure 1 [37]. After reading the complete articles, ultimately, five studies were included [37,38,39,40,41,42]. Two were eliminated as one did not use the radiologic imaging modality to identify the lesion but to measure its dimensions and the other established the diagnostic validity of ultrasound in comparison to histopathology and did not use radiographs for distinguishing the lesions [43,44].

Study characteristics regarding author details, imaging, accuracy, and outcome of the studies were identified and tabulated (Table 2). All five studies deemed the use of ultrasound as a more accurate and reliable method of distinguishing the nature of periapical lesions. Two studies showed a perfect agreement between the ultrasound and the reference test (histopathologic examination) [38,42]. Except for two studies [40,42], the remaining mentioned the characteristics of the observers who diagnosed the lesions. The ultrasonography image was interpreted by ultrasonographers while the radiographs were interpreted by endodontists or oral radiologists. All studies imaged lesions in the anterior areas of both the maxilla and the mandible. They used a linear probe for imaging, though the frequency of the waves used for imaging varied between studies. Two of the included studies did not specify the threshold criteria used for distinguishing lesions on the radiographs but mentioned that the observers were unable to do so [38,42].

According to the risk of bias tool (QUADAS—2), the presence of a high or an unclear risk or concern in any one domain classifies the study as “at risk of bias” or with “concerns regarding applicability”. The risk of bias tool indicated all studies at risk and having concerns for applicability. Table 3 summarizes the assessment. The selection of patients was a concern noted in all studies. The index test 1 (USG) and reference test (histopathology) had a low risk for bias or concern but the same could not be said for index test 2 (radiographs).

## 4. Discussion

This review compared the accuracy of ultrasound and conventional radiographic techniques in diagnosing periapical lesions. The five included studies that made this comparison found that using ultrasound for imaging provides the clinician with adequate information to accurately differentiate the nature of periapical lesions as cysts or granulomas. Ultrasound showed better accuracy in all studies despite the different criteria for radiographic classification used. However, all studies presented a risk of bias and concern regarding applicability according to the QUADAS—2 tool. Since the studies did not have a homogenous protocol for diagnosis of radiographic lesions and reporting was not uniform across them, a quantitative analysis was not possible.

Previous systematic reviews have studied the use of ultrasonography for diagnosing periapical lesions and compared it to histopathology [25,45]. They helped establish the diagnostic validity of ultrasonography for periapical lesions. However, like ultrasonography, histopathology—despite being the gold standard—is not a routine diagnostic procedure available to all. Though ultrasounds are less invasive, they also include the use of specialized equipment and training not generally provided to dental staff. To maximize their use in everyday practice, they must be compared to the current routine tests for periapical lesions—radiographs.

This study included clinical studies that compared conventionally used radiographs with ultrasonography to better understand the latter’s usefulness as a part of the dentist’s diagnostic toolkit. Periapical radiographs are used in routine dental practice to determine pulpal involvement in cases [46]. They have been used to determine the nature of the periapical lesion depending upon the size, shape, location, and effect on peripheral structures [17,47]. Ultrasound, on the other hand, is a newer imaging modality that will require training and investment from dentists to apply in clinical practice. This is evident in the studies that were examined. The ones that mentioned details of the observers, included a sonographer to analyze and diagnose USG imaging. The radiographs, however, were diagnosed by endodontists and oral radiologists. This helped blind the assessors of the index tests from each other to provide an unbiased diagnosis [48]. To truly compare the two tests, observers who are equally trained or experienced in the interpretation of both the tests, preferably dental professionals and blind to the cases being assessed with each index test, might make better examiners for deciding clinical applicability [49,50]. An observer who is proficient in identifying lesions on periapical radiographs and the ultrasound will help reduce bias arising due to operator skill. An assessor with a background in a single speciality assessing both images will help reduce bias due to specialization [51]. Blinding the assessor to the type of lesion and its diagnosis (from the gold standard test) will also help reduce detection bias [52].

Although CBCT is now used in almost all dental diagnoses, the studies in this review examined lesions in the anterior region only [49,50]. This region is the most common site for the occurrence of radiolucent lesions [50,51]. Additionally, alveolar bone thickness is known to increase from anterior to posterior regions [52,53]. Thus, it is more likely that lesions in the anterior regions of the jaws will cause erosion of the cortical plate, making the diagnosis of a lesion with ultrasound easier as it is believed that an intact cortical plate can hinder imaging [28]. Recent animal studies on the use of ultrasound demonstrate otherwise. A study on bovine mandibles indicated that lesions as small as 2 mm can be detected on ultrasound despite the presence of a cortical plate [54], indicating that it may be a useful technique for detecting small lesions that are present in the posterior regions as well.

The ultrasound probe was positioned extra-orally in all studies and some exclusively used it on the skin. It allowed visualization of the lesion, unlike radiographs that need a radiosensitive film placed intra-orally to provide a diagnosis. This is an advantage for diagnosis in cases where a severe infection or a large lesion does not allow adequate mouth opening in the patient or when a patient is not cooperative and cannot follow instructions. The placement of radiographic films in the posterior regions of the mouth often presents a problem where the films cannot be placed due to a shallow sulcus, which can be eliminated by this method [55,56,57].

Ultrasound imaging uses acoustic waves of a frequency higher than 20 KHz generated using magnetostriction or piezoelectric materials [58]. The differential impedance offered to these waves, by different tissues, reflects some waves back to the probe at their interface while some travel further. The portion of waves that are reflected back and the time it takes them to be reflected depends on the impedance contrast between the tissues and the distance of this interface from the probe. One of the earliest uses of ultrasonography in dentistry was in 1963 to examine tooth vitality [59]. Since then, ultrasound imaging has been used for various purposes in dentistry such as caries detection, muscle thickness, and temporomandibular disorders, among others [60]. However, the use of ultrasound in dentistry is still limited. When imaging biologic tissues, frequencies between 2–12 MHz are preferred. While low frequency provides a better penetration into tissues, a high frequency allows better spatial resolution and thus finer images. This is especially important when detecting lesions within the bone. The previously mentioned study, where lesions were detected within the periapical bone [56] used a frequency of 4.5 MHz compared to the studies included in this review where the range varied between 6–12 MHz.

In all studies that were included, the method of selecting patients was not reported. The criteria for inclusion and exclusion were delineated but the method of patient enrolment was not published. Thus, the potential for introducing a bias in patient selection was high. A study for diagnostic tests should either choose consecutive or random patients who are likely to have the condition. The aim is to include patients in a spectrum that is representative of patients seen in regular practice [61,62]. This is followed by applying the index and reference tests. In these studies, only patients who had lesions visible on the radiographs were included. Radiographs are used to detect the presence and also, absence of periapical lesions. None of the studies included a group who had symptoms of endodontic disease but no periapical lesions so that true specificity and sensitivity of the index tests could be determined. Another consideration for diagnostic studies is the calculation of a sample size using statistical principles to provide reliability to the study [63]. None of the studies here reported the use of statistical methods for sample size calculation. Both factors indicate caution while interpreting the results of these comparative studies.

Future studies with a pre-determined protocol to reduce the risk of bias, as seen here due to patient selection, are needed. Sample size calculation to promote reliability is also a must. Studies also need to focus on other areas of the mouth for diagnosis of periapical lesions or their absence. This can be done by including a spectrum of patients that is similar to everyday practice in these studies. Recently, the use of artificial intelligence to interpret findings in maxillofacial radiology has also been done to eliminate human error and provide better diagnoses [63,64,65]. Similar systems can also be developed for ultrasound imaging to provide an accurate, non-invasive diagnosis to patients.

## 5. Conclusions

Ultrasound offers a non-invasive, radiation-free method for detecting periapical lesions in the jaws. Within the limitations of the studies included, this review indicates that it provides better diagnostic accuracy for differentiating endodontic lesions compared to radiographic imaging. Current studies provide a very limited picture of the utility of this imaging method in diagnosing periapical lesions. There is a need for further diagnostic studies with rigorous protocols and designs that help minimize bias and increase the reliability of the outcome. Studies also need to compare ultrasound imaging to CBCT which is a more sensitive imaging method for periapical lesions than radiographs and is considered the current gold standard for periapical imaging.

## Figures and Tables

**Table 1:** Search queries used in different databases.

Database	Search Details	Results
PubMed	(“periapical” [All Fields] OR “periapically” [All Fields] OR “periapicals” [All Fields]) AND (“lesion” [All Fields] OR “lesion s” [All Fields] OR “lesional” [All Fields] OR “lesions” [All Fields]) AND “diagnos*” [All Fields] AND (“ultrasonography” [MeSH Terms] OR “ultrasonography” [All Fields] OR (“ultrasound”[All Fields] AND “imaging”[All Fields]) OR “ultrasound imaging” [All Fields]) AND (“diagnostic imaging” [MeSH Subheading] OR (“diagnostic” [All Fields] AND “imaging” [All Fields]) OR “diagnostic imaging” [All Fields] OR “radiography” [All Fields] OR “radiography” [MeSH Terms] OR “radiographies” [All Fields] OR “radiographys” [All Fields])	36
Scopus	TITLE-ABS-KEY (periapical AND lesion AND ultrasound AND radiography)	22
Web of Science	(periapical (ultrasound or echography) radiograph)	17
Embase	‘periapical ultrasound radiograph’ OR (periapical AND (‘ultrasound’/exp OR ultrasound) AND (‘radiograph’/exp OR radiograph))	12

**Table 2:** Characteristics of selected studies.

Sno.	Author/Year, Country	Sample Size	Age	Ultrasound			Radiographs		Outcome
				Machine	Frequency	Accuracy	Imaging Modality	Accuracy	
1	Gundappa et al./2006, UK	15	13–40 years	LOGIQ-500 PRO (GE Medical System, USA), with color Doppler	8–11 MHz used extra- and intra-orally	100%	Conventional and digital IOPAs	Unable to differentiate with conventional or digital radiography	While USG underestimates the dimensions of a lesion, it is more accurate in diagnosing the nature of the lesion which is not possible with radiographs in the anterior region of the jaws
2	Raghav et al./2010, India	21	15–45 years	Voluson 730 Pro Machine (GE Medical Systems) with color Doppler	8–11 MHz used extra-orally	95.20%	Conventional and digital IOPAs	47.6% (conventional) and 55.6% (digital)	USG is a reliable diagnostic technique to distinguish between the nature of periapical lesions in the anterior region of the jaws compared to radiographic imaging
3	Goel et al./2011, India	30	15–50 years	SonoAce 8000 Live^®^ machine (Medison, Seoul, Korea)	9 MHz extra- and intra-orally	96.67% (C) and 96.67% (G)	Conventional IOPAs	66.67% (C) and 66.67% (G)	USG is superior to intra-oral radiographs inaccurate diagnosis of the nature of periapical lesions in anterior regions of the jaws
4	Sandhu et al./2015, India	30	15–50 years	Volusion-730- expert (GE Medical System, USA), with color Doppler	6–12 MHz used extra-orally	16/16 (G) C—not reported	Conventional and digital IOPAs	11/16 (G) C—not reported	Radiographic imaging only provides information about the presence or absence of a periapical lesion, their nature, however, especially of mixed lesions can only be diagnosed with USG
5	Khambete and Kumar/2015, India	10	19–40 years	LOGIQ-500 PRO (GE Medical System, USA), with color Doppler function	8–11 MHz used extra-orally	100%	Conventional IOPAs	Unable to differentiate with conventional or digital radiography	USG can be used as an adjunct for imaging and diagnosis of periapical lesions and is a valuable tool in the radiation-free non-invasive diagnosis of these lesions

C—cyst, G—granuloma, USG—Ultrasonography.

**Table 3:** Summary of Quality Assessment of Studies.

SNo.	Author/Year, Country	Risk of Bias					Applicability Concern			
		Patient Selection	Index Test 1 (USG)	Index Test 2 (Radiograph)	Reference Test	Flow and Timing	Patient Selection	Index Test 1	Index Test 2	Reference Test
1	Gundappa et al./2006, UK	H	L	U	L	L	H	L	U	L
2	Raghav et al./2010, India	H	L	L	L	L	H	L	L	L
3	Goel et al./2011, India	H	L	L	L	L	H	L	L	L
4	Sandhu et al./2015, India	H	L	L	L	L	H	L	L	L
5	Khambete and Kumar/2015, India	H	L	U	L	L	H	L	U	L

H—high, U—unclear, L—low.

## Data Availability

Not applicable.
